# Ulnar shortening osteotomy for ulna impaction syndrome with positive ulnar variance: retrospective outcome analysis

**DOI:** 10.2340/17453674.2025.43086

**Published:** 2025-03-10

**Authors:** Marisa VALENTINI, Eva KALCHER, Silvia ZÖTSCH, Andreas LEITHNER, Philipp LANZ

**Affiliations:** Department of Orthopedics and Trauma, Medical University of Graz, Austria

## Abstract

**Background and purpose:**

We primarily aimed to report the results of ulnar shortening osteotomy (USO) in cases of ulna impaction syndrome (UIS), and secondarily to assess the influence of etiology, radiographic parameters, and comorbidities on the outcome.

**Methods:**

Patients with USO performed for UIS between 2014 and 2022 at our department were included in the study. Demographic, surgical, and postoperative data, including complications and revisions, were recorded retrospectively. An additional study-specific follow-up was performed in all available cases, including subjective outcome measures as Patient Related Wrist Evaluation (PRWE) and Quick Disability of the Arm Shoulder and Hand (Quick-DASH) scores, and standardized 90–90° wrist radiographs.

**Results:**

47 patients were treated with USO at mean age 45.8 years (standard deviation [SD] 16.7); 28 were female; median follow-up was 37 months (interquartile range [IQR] 22–57). Isolated USO was performed in 27 cases; the rest received a combination of procedures, e.g., wrist arthroscopy. USO-specific devices were used in all cases. Reoperations were performed in 12 cases, with implant removal in 11. Postoperative complications such as chronic regional pain syndrome or pseudoarthrosis were detected in 9 patients. 29 patients were additionally examined at median 36 months (IQR 22–49) follow-up. A median PRWE score of 7 (IQR 0–19) and a median Quick-DASH score of 4.5 (IQR 0–15.9) were reported. The subjective improvement was rated as very high by 24 patients. Radiographs showed a mean ulnar shortening of 2.9 mm (SD 1.1) and bone consolidation was achieved in all osteotomies at last follow-up. Relevant comorbidities weakly correlated with worse outcome scores (ρ = 0.41, 95% confidence interval [CI] –0.05 to 0.74 for PRWE and ρ = 0.40, CI –0.06 to 0.73 for Quick-DASH). No statistically significant difference could be detected in any other variables, including UIS etiology.

**Conclusion:**

We found that USO had good subjective results measure scores, but with relatively high complication and revision rates, including implant removal.

Ulna impaction syndrome (UIS) is a common degenerative condition due to biomechanical changes with chronic excessive loading across the ulnocarpal joint [1,2]. UIS is associated with a spectrum of pathological changes involving the triangular fibrocartilage complex (TFCC) and articular surfaces of the ulnar head, lunate, and triquetrum, as well as lunotriquetral ligament tears [2-4]. It mainly affects patients with positive ulnar variance (PUV), as an increase of the ulnar length directly correlates with an increase of the ulnar load [1,5]. Only when symptomatic is PUV to be considered pathological (as ulnar variance is normally distributed in the general population) [1,2]. UIS and PUV can be primary (idiopathic) or secondary (posttraumatic, e.g., after distal radius fractures) [2,6,7]. UIS is partnered with a variety of symptoms, including ulnar-sided wrist pain, impaired grip strength or wrist range of motion, often leading to upper extremity disability, and limitations in daily life and work ability [2,8]. Therapeutic options range from nonoperative management (initial therapy in most cases) to surgical treatments [2,5,8]. Surgical treatment can be considered when nonoperative management is insufficient or non-effective. One surgical option is the ulnar shortening osteotomy (USO), aiming to decompress the load on the ulnocarpal joint by shortening the ulna and therefore correcting the PUV [9,10]. Another surgical treatment alternative is the arthroscopic wafer procedure (distal ulna resection) [11].

Previous studies have evaluated the effectiveness of USO, showing good subjective results and beneficial outcomes, as well as a relatively high number of complications [12-17]. Described complications following USO included nonunion and the need for plate removal due to irritation [17-19]. Chan et al. [17] summarized the prevalence of complications and revision rates across studies finding large variations; implant removal rates ranged from 0% to 45%. The influence of UIS etiology on treatment outcomes remains unclear throughout the literature thus far.

We primarily aimed to report the results of USO for UIS by retrospectively analyzing all cases operated on in our department, and secondarily to assess the influence of various variables such as etiology, radiographic parameters, and comorbidities on the outcome.

## Methods

### Study design

All patients treated with USO for UIS between 2014 and 2022 at the Orthopedics and Trauma department of the Medical University of Graz, Austria, were included and retrospectively analyzed. The study is reported according to STROBE guidelines.

### Population

**Figure 1:**
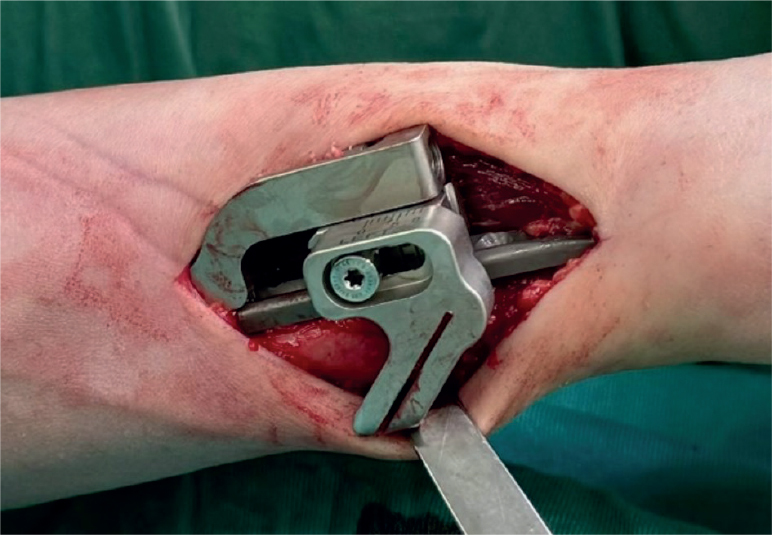
Intraoperative view of an ulnar shortening osteotomy specific implant: ulnar plate (volarly placed), and the plate-associated device for ulnar shortening osteotomy and compression.

Case history and clinical follow-up were retrieved from the hospital internal data systems, also including data from other regional public hospitals (minimizing the chance of undetected revision surgeries). Follow-up time was calculated from the index operation to the last follow-up date. The USO surgery indication was established after extensive diagnostics, unsuccessful nonoperative treatment, and patient information. All patients received magnetic resonance imaging of the wrist prior to surgical intervention; in cases of operable TFCC lesions the surgery was combined with wrist arthroscopy to address the defect or tear in the same operation. In all patients an edema zone was radiologically confirmed at the height of the TFCC (increased TFCC signal). Surgical as well as peri- and postoperative treatment was standardized. All USOs were executed by 2 experienced surgeons (level 3 of expertise, Tang & Giddin’s criteria [20]). All surgeries were performed with a USO-specific device. All plates were positioned volarly on the middle to distal ulnar diaphysis, and fixated by means of at least 3 screws per segment. An intraoperative picture of a USO-specific implant is shown in Figure 1. Wrist splinting was applied for 2 weeks postoperatively. Hand therapy and finger mobilization was started on day 1, physiotherapy including free wrist range of motion on week 3. Strengthening and weightbearing exercises were not allowed for 6 weeks postoperatively, or maximal loading and sport for 3 months. Standard follow-ups were scheduled at 2 and 6 weeks, 3 months, and 1 year postoperatively. All included cases were screened for peri- and postoperative complications, as well as for the need or wish for implant removal due to disturbing or painful plate situs. Chronic regional pain syndrome (CRPS) was diagnosed according to international guidelines and confirmed as a complication through specialist consultation; pseudarthrosis was defined as non-union 6 months postoperatively. All patients were reached by phone and the documented complications and revisions confirmed.

A study-related follow-up at our department, including the standardized radiographs and the patient-related outcome measure (PROM) scores, was performed with the available patients. Variables possibly influencing the outcome (e.g., dominance of the operated hand, UIS etiology) were recorded. The patients were screened for relevant comorbidities: impairing rheumatological, psychiatric, or musculoskeletal diseases, as well as previous operations on the ipsilateral wrist were included as relevant medical history.

### Radiography

**Figure 2:**
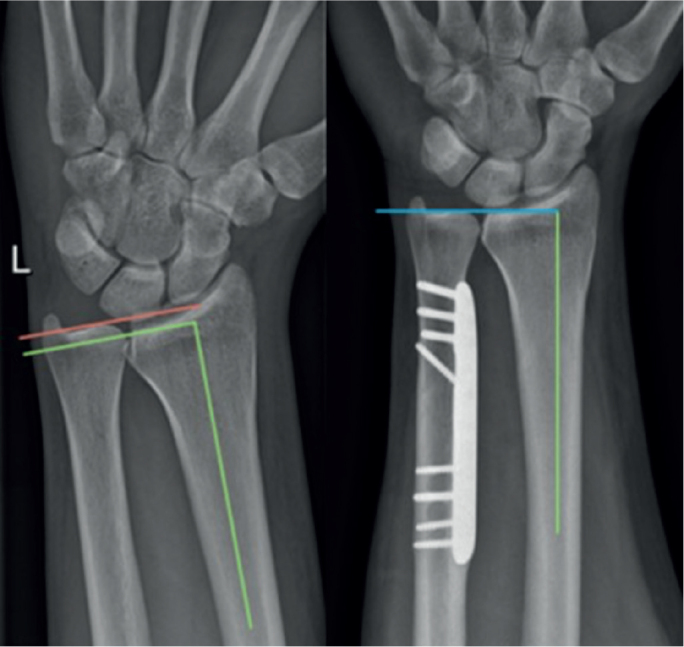
Ulnar variance measurements on the 90–90° wrist radiographs preoperatively (positive ulnar variance, left panel) and postoperatively (neutral ulnar variance, right panel).

A bilateral so-called 90–90° wrist radiograph was taken preoperatively and postoperatively (at last follow-up). This radiograph is taken with the shoulder positioned in 90° of abduction and the elbow flexed to 90°, while the forearm is placed in mid-pronation. The ulnar variance was measured radiographically by defining the perpendicular line to the radius axis through the radio- and ulnocarpal articular surface at the level of the distal radioulnar joint, and comparing their lengths (Figure 2). The radiograph itself and the measurements were standardized and reproducible (all measurements were done by and compared between 2 independent observers). The following features were radiographically assessed: (i) positive ulnar variance preoperatively (mm), (ii) ulnar variance difference from the contralateral side preoperatively (mm), (iii) ulnar variance postoperatively (mm), (iv) ulnar shortening postoperatively versus preoperatively (mm), and (v) bone consolidation.

### Outcome measures

Subjective improvement after the performed surgery was measured through a 5-point Likert scale: much better (5 points), better (4), same (3), worse (2), much worse (1). At last follow-up the wrist range of motion (ROM) was evaluated. As PROMs, the Patient Related Wrist Evaluation score (PRWE) and the Quick Disability of the Arm Shoulder and Hand score (Quick DASH) were chosen.

### Statistics

Statistical analyses were performed with SPSS Statistics (Version 28 for Windows; IBM Corp, Armonk, NY, USA). Continuous variables were checked for normality with the Shapiro–Wilk test. Median values with the respective interquartile ranges (IQR) were reported for non-normally distributed variables, whilst mean values with respective standard deviation (SD) were registered for normally distributed variables. Regarding the follow-up data retrieved for the total population, with no systematic differences between the missing and observed values, the unavailable data can be considered “missing completely at random” [21]. The outcome results were analyzed considering different samples or subgroups to evaluate the influence of each factor through nonparametric independent-samples Mann–Whitney U test. Correlations between the outcome measures and continuous variables factors were tested through Spearman’s correlation test, with 95% confidence interval (CI).

### Ethics, data sharing plan, funding, use of AI, and disclosures

Written informed consent was obtained from all included subjects before the study. The current study was approved by the local institutional review board (IRB number: 35-031 ex 22/23). The datasets used and/or analyzed during the current study are available from the corresponding author on reasonable request. This study did not receive any funding. AI was not used. All authors declare no competing interests related to the research, authorship, and/or publication of this article. Potential competing interests, unrelated to this research/publication, are as follows. MV received travel support from Alphamed, outside the submitted work. PL and SZ received payment and endorsement for congress and courses presentations by Nuvasive, outside the submitted work. AL received institutional educational grants by Johnson & Johnson, Alphamed, and Medacta, outside of the submitted work. Complete disclosure of interest forms according to ICMJE are available on the article page, doi: 10.2340/17453674.2025.43086

## Results

### Population

**Figure 3:**
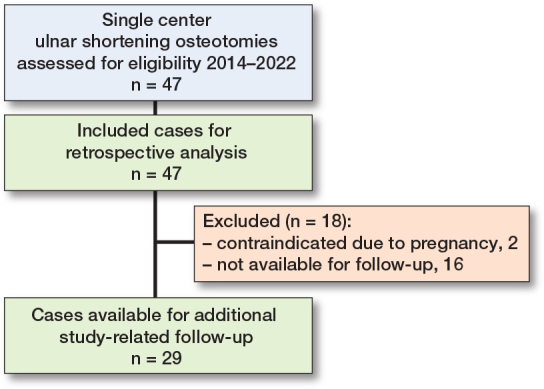
Flow diagram of the inclusion process of the study population.

47 USO cases were included (Figure 3 and Table 1). The average age at index operation was 45.8 years (range 20–77; SD 16.7). Female sex was dominant (28 women and 19 men). The median follow-up was 37 months (range 10–111; IQR 22–57). An isolated USO was performed in 27 cases, the rest received a combination of procedures (e.g., USO and wrist arthroscopy). 4 types of USO-specific devices/implants were used: 42 Ulna Osteotomy Locking Plate (ITS, Graz, Austria), 2 APTUS Ulna Shortening System (Medartis, Basel, Switzerland), 2 LCP Ulna Osteotomy System (Depuy Synthes, Johnson & Johnson, New Brunswick, NJ, USA), 1 RECOS Ulna Shortening Plate (KLS Martin, Freiburg, Germany).

**Table 1:** Descriptive analysis of patient cohort (N = 47)

Variable	
Mean age at surgery, years (SD)	45.8 (16.7)
Sex (male/ female)	19/28
Surgery	
Isolated USO	27
Combination of USO and other forearm procedures	20
wrist arthroscopy	7
removal of prior implants	6
removal of avulsion fragments	3
others **^a^**	4
Follow-up, months, median (IQR)	37 (22–57)
Postoperative complication rate	9
CRPS	2
Implant loosening	2
Pseudoarthrosis	2
Delayed union	1
Lesion dorsal branch of the ulnar nerve	1
Suture granuloma	1
Reoperation rate	12
Implant removal	11

SD = standard deviation; USO = ulnar shortening osteotomy; IQR = interquartile range; CRPS = chronic regional pain syndrome.

a First extensor tendon compartment release (1), wrist resection arthroplasty (1), radioscapholunate fusion (1), and elbow arthroscopy (1).

### Complication and revision rates

**Figure 4:**
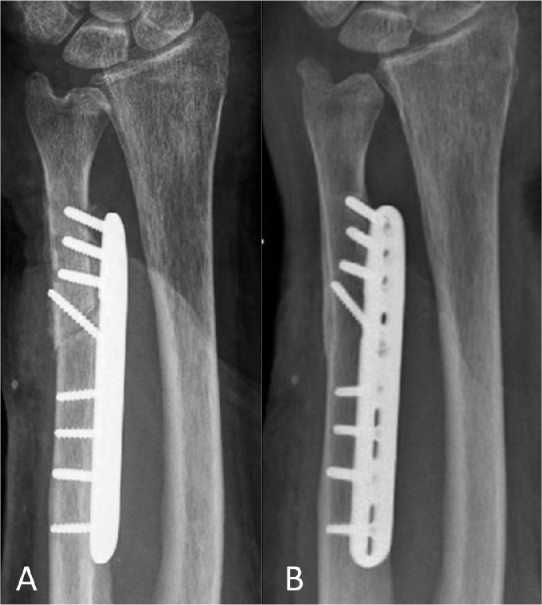
Asymptomatic loosening case: radiographs (A) 6 weeks postoperatively and (B) at last follow-up 16 months postoperatively. No relevant trauma or infection could be identified as cause for the loosening; the patient remained asymptomatic throughout the 16 months follow-up.

**Figure 5:**
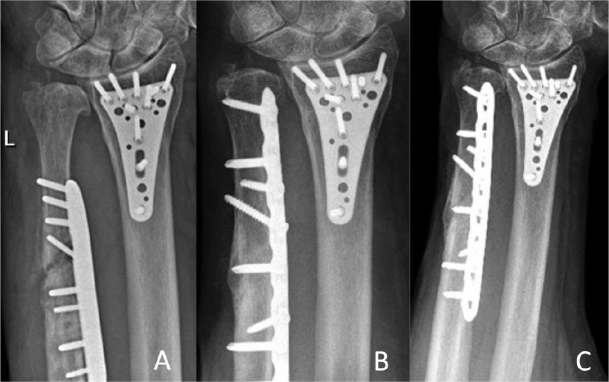
Posttraumatic loosening case. Radiographs (A) 2 months postoperatively with plate loosening after fall on the ipsilateral hand; (B) 12 months after the revision-osteosynthesis; and (C) at last follow-up 36 months postoperatively.

**Figure 6:**
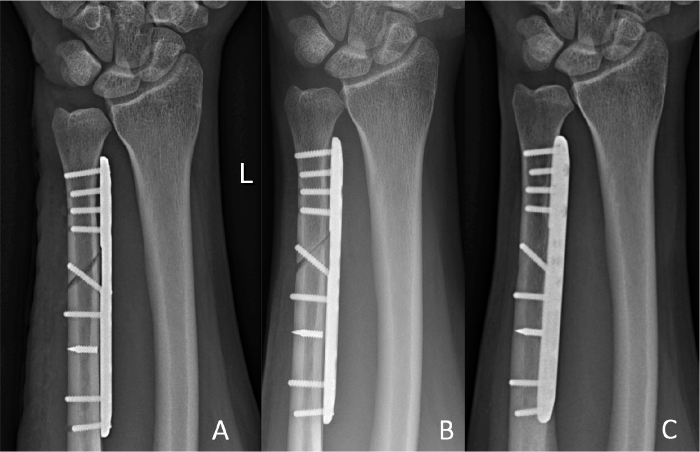
Pseudoarthrosis case. Radiographs (A) 2 weeks postoperatively; (B) 6 months postoperatively (pseudoarthrosis before undergoing ESWT at 6.5 months follow-up); and (C) 12 months postoperatively.

Implant removal was performed in 11 of the 47 cases. 9 postoperative complications were detected: 2 CRPS, 2 implant loosenings (1 posttraumatic that underwent successful revision, 1 asymptomatic requiring no further therapy), 2 pseudarthroses (successfully treated with extra-corporal shock-wave therapy (ESWT), resolving symptoms and achieving union), 1 delayed union (no further intervention needed, achieving consolidation at 4 months’ follow-up), 1 iatrogenic axonal lesion of the dorsal branch of the ulnar nerve, and 1 suture granuloma (successfully surgically removed at 2 months follow-up). Therefore, a cumulative overall reoperation rate of 12/47 was observed (implant removal was later performed in the suture granuloma case). 3 of the cases that developed complications are illustrated in Figures 4–6 (see Appendix).

### Outcome measures

29 patients were further examined through a study-specific follow-up (Figure 3 and Table 2). 18 patients (2 contraindicated due to pregnancy, 16 unavailable) had to be excluded from further examination, due to the unavailability for the study-related follow-up at our department. At a median follow-up of 36 months (range 10–95; IQR 22–49), 18 women and 11 men were clinically and radiographically examined. The mean age at index operation was 49.6 years (range 23–77; SD 16.0). 12 of the operated-on hands were dominant. More than half of the patients suffered a primary idiopathic UIS, while 12 cases were secondary posttraumatic UIS. USO was performed as a stand-alone operation in 17 cases, while 12 patients received a combination of procedures: USO as well as another operation involving the ipsilateral forearm. For example, wrist arthroscopy was carried out in 7 cases and removal of symptomatic posttraumatic avulsion fragments of the ulnar styloid process in 3. The patients were screened for relevant comorbidities or medical history (n = 8). At last follow-up the median PRWE score was 7 (range 0–43.5; IQR 0–19) and the median Quick-DASH score 4.5 (range 0–56.8; IQR 0–15.9). The subjective improvement was rated as very high by 24 patients (“much better,” 5 points on the 5-point Likert scale), 4 assessed the improvement as good (“better,” 4 points), and only 1 patient, who developed postoperative CRPS, did not report any improvement through the procedure (“worse,” 2 points). At clinical examination the wrist ROM was painful in 4 and limited in 6 patients with 3 of these cases overlapping (ROM both limited and painful), leading to a total of 7 patients reporting an impaired ROM at last follow-up. Radiographically, an average ulnar shortening of 2.9 mm was achieved (range 1.0–5.5; SD 1.1). The median preoperative PUV was 2.9 mm (range 0–9.9; IQR 1.9–4.3). The average PUV difference from the contralateral side was 2.1 mm (range 0–5.5; SD 1.4). The median postoperative PUV was 0.1 mm (range –0.9 to 4.4; IQR 0–1.1). All osteotomies were consolidated at last follow-up.

**Table 2:** Descriptive analysis of further examined patient cohort (n = 29)

Variable	
Mean age at surgery, years (SD)	49.6 (16.0)
Sex (male/ female)	11/18
Surgery	
Isolated USO	17
Combination of USO and other procedures	12
Follow-up, months, median (IQR)	36 (22–49)
Dominance of operated on hand (dominant/non-dominant)	12/17
UIS etiology (idiopathic/secondary)	7/12
Relevant comorbidities (yes/no)	8/21
PRWE score, median (IQR)	7 (0–19)
Quick DASH score, median (IQR)	4.5 (0–15.9)
Subjective improvement	
Much better	24
Better	4
Worse	1
Radiographic parameters	
Preoperative PUV, mm, median (IQR)	2.9 (1.9–4.3)
Ulnar shortening, mm, mean (SD)	2.9 (1.1)
Bone consolidation at last follow-up	100%

For abbreviations, see Table 1 and UIS = ulna impaction syndrome; PRWE = Patient Related Wrist Evaluation score; Quick DASH = Quick Disability of the Arm Shoulder and Hand score; PUV = positive ulnar variance.

Relevant comorbidities and medical history seem to correlate with worse outcome in terms of PROMs (PRWE and Quick DASH), without reaching statistical significance: Spearman correlation coefficient ρ = 0.40, CI –0.05 to 0.74 for Quick DASH and ρ = 0.41, CI –0.06 to 0.73 for PRWE (moderate correlations with non-significant CIs). Also, all other comparisons between subgroups or variables were found to be non-significant in the statistical evaluations (Table 3). No influence of sex on the results could be detected either. Having operation on the dominant hand (rather than the non-dominant), having secondary UIS (not primary), and receiving a combination of forearm procedures (instead of USO alone) are weakly correlated with worse subjective outcome (PROMs). Furthermore, continuous variables such as age, follow-up time, ulnar shortening length, and preoperative PUV were tested for correlation with the outcome measures. All correlations were non-significant and very weak (ρ < 0.20). Larger shortenings weakly correlate with worse results, whilst elevated preoperative PUV measurements weakly correlate with lower scores (i.e., better results). Both PROMs had a weak negative correlation with age: as age at index operation increases, the scores decrease (the outcome improves). The same was observed regarding follow-up time: as the follow-up increases, the scores decrease (the outcome measures improves).

**Table 3:** Statistical results through Spearman’s correlation test. Values are Spearman’s rho with (95% confidence interval)

Variables	PRWE	Quick DASH
Dominant vs non-dominant hand	0.09 (–0.30 to 0.45)	0.04 (–0.35 to 0.40)
Secondary vs idiopathicUIS	0.02 (–0.38 to 0.40)	0.17 (–0.27 to 0.55)
Isolated USO vs combined procedures	–0.11 (–0.48 to 0.26)	–0.02 (–0.40 to 0.37)
Relevant comorbidities vs none	0.41 (–0.06 to 0.73)	0.40 (–0.05 to 0.74)
Ulnar shortening, mm	0.02 (–0.43 to 0.38)	0.03 (–0.42 to 0.42)
Preoperative PUV, mm	–0.00 (–0.46 to 0.41)	–0.00 (–0.41 to 0.40)
Age, years	–0.12 (–0.51 to 0.30)	–0.13 (–0.51 to 0.29)
Follow-up, months	–0.05 (–0.43 to 0.35)	–0.05 (–0.35 to 0.44)

For abbreviations, see Table 2.

## Discussion

We primarily aimed to evaluate the results of USO in patients with UIS, and secondarily to assess the influence of various variables such as etiology, radiographic parameters, and comorbidities on the outcome.We found that USO can be an effective surgical procedure with good subjective results, although with high complication and reoperation rates in accordance with the published literature [16-19,22].

The important role of ulnar variance in UIS has long been known. Palmer and Werner [5] demonstrated the direct relationship between increasing ulnar length and force transmission across the TFCC or ulnocarpal joint. Ulnar neutral wrists transfer approximately 18% of the total load, with the radiocarpal joint transferring 82% of the total force [5]. A positive ulnar variance of 2 mm will increase the ulnocarpal load to approximately 40% and increased dorsal tilt due to previous injuries (distal radius fractures) can additionally increase the ulnar load to 65% of total force transferred [5]. UIS with PUV comprise the selected cases where ulnar shortening osteotomy can be effective. Our study describes the standardized 90–90° wrist radiograph and its possible important use in evaluating and measuring the ulnar variance. While the pronated grip view, popularized by Tomaino [23], allows depiction of dynamic ulnar impaction and load, this standardized radiograph (unloaded) allows a precise and replicable measurement and comparison of ulnar variance.

The outcome results assessed through PROMs (PRWE and Quick DASH scores) and subjective improvement (5-point Likert scale) were very good, suggesting overall patient satisfaction. The values are in accordance with previous literature [16-19,22].

By comparing the outcome measures between subgroups, we found a noteworthy difference based on only 1 variable: relevant comorbidities and medical history. We observed a significant P-value (< 0.05), with non-significant moderate correlation and wide range of the CI, which the small sample size and high data variability may account for. This reflects common expectations: relevant and impairing rheumatological, psychiatric, or musculoskeletal diseases, as well as previous operations on the ipsilateral wrist, are bound to badly influence the overall outcome. Until now, this variable had not been included in any previous study or statistical examination. Although no statistical significance was detected (non-significant effect estimates with 95% confidence intervals), physicians should include in their decision-making process their patients’ comorbidities, in order to advise properly on treatment.

Age, follow-up time, ulnar shortening length, preoperative positive ulnar variance, as well as for all other subgroups (dominant vs non-dominant hand, idiopathic vs secondary UIS and PUV, isolated USO vs combination of procedures), did not significantly influence the results. Larger shortening weakly correlated with worse results, suggesting that excessive shortening could possibly cause pain, impaired function, or symptoms. As age at index operation increases, the outcome improves; the reason for this finding might be the reduced functional requirements of older patients, whilst slight impairments in wrist function can strongly negatively influence life and work of younger individuals. As expected, as follow-up time increases, the outcome improves. Dominance of the operated hand, secondary UIS etiology, and receiving a combination of procedures with USO may be associated with worse outcome, although not reaching statistical significance. To our knowledge, this is the first study aiming to systematically assess the possible correlation or influence of these factors on the operation’s outcome.

Chan et al. [17] summarized the prevalence of complications across studies and found large variations, highlighting the morbidity associated with ulnar shortening osteotomy. A 6.3% risk of nonunion and 45% plate removal rate due to mechanical irritation was detected for this procedure [17]. The authors concluded that surgeons should consider the high complication and metal irritation rates and counsel patients appropriately when offering USO, in particular the likelihood of the need for plate removal [17]. Similarly, Teunissen et al. [16] analyzed a cohort of 106 patients who received USO, finding beneficial outcomes, although there was a large variance and a relatively high number of complications. Out of the total 106 cases, 44 had secondary UIS after distal radius fractures, and 64% of patients experienced at least 1 complication [16]. Mechanical irritation and plate removal rates were, respectively, 47% and 32%. Nonunion was detected in 6% of cases [16]. These results are in line with those of our study, with implant removal and pseudoarthrosis rates of 11/47 and 2/47 respectively.

An important factor to be borne in mind is the plate positioning. This might greatly influence the outcome, especially considering the high mechanical irritation and implant removal rates found. All plates in this study were positioned volarly on the middle to distal ulnar diaphysis, to achieve maximal possible soft tissue coverage, aiming to prevent mechanical irritation and avoid disturbing or painful localizations. This might have contributed to the slightly lower removal rates in comparison with previous studies [16,17]. This aspect has not yet been analyzed in the available literature; further studies specifically comparing different implant positionings would be necessary to prove the potential relevance of this aspect.

It is worth mentioning that, when dealing with posttraumatic secondary UIS and PUV, surgeons must consider correction of the radial deformity as an alternative to USO. The authors’ choice to perform ulnar shortening rather than intervening on the shortened distal radius in these posttraumatic cases was based on the following factors: surgical technique through the USO-specific devices and implants, surgical approach, preoperative clinic attendance with isolated ulnar-sided wrist pain and the degree of distal radius deformity. Nevertheless, in cases of relevant distal radius deformity, isolated USO should not be the surgery of choice (rather a combination of deformities’ correction, as reported for example by Gogna et al. [24]). Another operative option in cases of UIS with PUV (idiopathic or secondary) is the arthroscopic wafer procedure (distal ulna resection) [11,25]. Yu et al. [25] systematically reviewed the available literature to compare this procedure with USO, finding good results for both techniques, but an advantage of USO in terms of results when treating pronounced PUV.

### Limitations

First, only 29 of 47 performed USO could be assessed through PROMS, radiographically and clinically at last follow-up, causing missing outcome data for the rest of the patients and potential bias. Although functional and subjective outcome measures are not generalizable to the entire cohort, retrospective data including complications could be collected for all cases, allowing an appropriate analysis of the complication, revision, and implant removal rates. Also, the demographic data for the overall population and the follow-up cohort are comparable. The non-available data can be assumed to be “‘Missing Completely At Random” [21]. Second, a major part of the collected data was gathered retrospectively, relying on electronic patient dossiers. Third, while all USOs were performed at the level of the diaphysis using an oblique cut through a specific USO device, different implants were used. This variability might have influenced the outcome. A direct comparison between the different devices was not the intention of the authors and would not have been possible due to the unequal distribution, with the majority of cases being operated on with 1 specific implant. Previous research did not find a significant difference between freehand USO and the use of specific USO devices [18,22], suggesting that there might also not be a difference between different USO implants. Fourth, due to this study’s retrospective nature there were no preoperative PROMs. The direct comparison and assessment of improvement of the scores between preoperative and postoperative could have delivered more insightful information (especially considering the variable “relevant comorbidities”). Fifth, the study sample was not homogeneous regarding other factors or variables that may have influenced the surgical outcome. For example, some patients underwent concomitant surgery with USO, which could have induced some co-treatment bias, although this is difficult to generalize for different types of procedures. Nonetheless, this study achieves comparison of outcome measures between isolated USO and USO performed in combination with other procedures on the ipsilateral forearm. Sixth, a wide range of comorbidities were grouped together, although each condition might have very different influences on wrist function and postoperative outcome. Due to the limited cohort size, it was not possible or productive to further categorize the registered comorbidities.

### Conclusion

We found that USO had good subjective results, although with high complication and reoperation rates, including implant removal. In perspective, USO can be an effective surgical procedure for UIS with PUV; the results of this study may be used in preoperative patient counseling and shared decision-making processes.

## Notes

MV designed the study, reviewed electronic patient dossiers, performed the analysis, discussed the results, and wrote and reviewed the manuscript. EK reviewed electronic patient dossiers, performed the analysis, and reviewed the manuscript. SZ designed the study, discussed the results, and reviewed the manuscript. AL discussed the results and reviewed the manuscript. PL designed the study, discussed the results, and reviewed the manuscript. All authors reviewed and edited the manuscript. All authors read and approved the final manuscript.

The authors thank all patients who participated and allowed their data to be anonymously used for the present study.

Handling co-editors: Jeppe Vejlgaard Rasmussen and Robin Christensen

*Acta* thanks Nickolaos A Darlis and Robert Gvozdenovic for help with peer review of this manuscript.
